# Independent and combined effects of smoking, drinking and depression on periodontal disease

**DOI:** 10.1186/s12903-024-04287-6

**Published:** 2024-05-06

**Authors:** Y. Q. Huang, J. N. Xu, Y. Huang, Y. D. Xu, H. L. Wang, W. T. Shi, J. Wang, H. Wang

**Affiliations:** 1 School of Public Health, Shanghai Jiao Tong University School of Medicine, 227 Chongqing Road, Huangpu District, Shanghai, China; 2 Department of Prosthodontics, Shanghai Ninth People’s Hospital, College of Stomatology, Shanghai Jiao Tong University School of Medicine, No.639 Zhizaoju Road, Huangpu District, Shanghai, 200011 China; 3 National Center for Stomatology, National Clinical Research Center for Oral Diseases, Shanghai Key Laboratory of Stomatology College of Stomatology, Shanghai Jiao Tong University, Shanghai Research Institute of Stomatology, Shanghai, China; 4 Nanjing Medical University, Nanjing, China; 5 Clinical Research Unit, Shanghai Ninth People’s Hospital, Shanghai Jiao Tong University School of Medicine, Shanghai, China

**Keywords:** Periodontitis, Depression, Smoking, Drinking, Interactive effect, NHANES

## Abstract

**Background:**

Periodontitis is a complex chronic inflammatory disease that is particularly associated with health-related conditions such as smoking, excessive drinking and depression. This research aimed to investigate the interaction between these lifestyles factors on periodontitis risk.

**Methods:**

This study included participants who participated in the National Health and Nutrition Examination Survey in the United States between 2009 and 2014. They had completed oral health-periodontal examination, Smoking-Cigarette Use Questionnaire, Alcohol Use Questionnaire, and Patient Health Questionnaire. Periodontal clinical attachment loss (CAL) of 3 mm or more and Patient Health Questionnaire-9 (PHQ-9) of 10 scores or more were used to identify periodontitis and depression, respectively. Daily alcohol consumption in the past year was classified into three levels: low (1 drink or less), moderate (between 1 and 3 drinks), and heavy drinking (4 drinks or more), while smoking was defined as having smoked at least 100 cigarettes in one's lifetime. Then, the logistic regression combined with interaction models were used to analyze the independent and combined effects of smoking, drinking and depression on periodontitis risk.

**Results:**

The results indicated a statistically significant multiplicative interaction between smoking and depression in relation to the development of periodontitis, both in the overall population (*P* = 0.03) and among male participants (*P* = 0.03). Furthermore, among individuals experiencing depression, smoking was found to significantly increase the prevalence of periodontitis by 129% in the younger age group compared to non-smokers (odds ratio [OR]: 2.29; 95% confidence interval [CI]: 1.10 to 4.76). However, the interaction between smoking and alcohol consumption was only significant among females (*P* < 0.05). There was a dose-dependent relationship between drinking frequency and smoking on periodontitis prevalence. In the smoking population, occasional drinking (OR: 1.70; 95% CI: 1.22 to 2.37) and regular drinking (OR: 2.28; 95% CI: 1.68 to 3.11) significantly increased the prevalence of periodontitis compared to individuals without these two factors.

**Conclusion:**

These results suggested that there were interactive effects between smoking, drinking and depression on periodontitis risk and policies aimed at healthy behaviours and mental health may be beneficial for our oral health.

**Supplementary Information:**

The online version contains supplementary material available at 10.1186/s12903-024-04287-6.

## Background

Periodontitis is a common chronic inflammatory disease with a high incidence of 50% [1], which increases the risk of systemic diseases, such as diabetes, cardiovascular diseases, Alzheimer's disease, and mental disorders [2, 3]. The important role of inflammatory bimarkers, such as cortisol, TGF-1β and TNF-α, in the pathogenesis of periodontitis has drawn the attention to the systemic impact of periodontitis and its potential relationship with other diseases [4, 5]. More recently, psychological conditions, most notably depression, have been shown to be associated with periodontitis. According to the Global Burden of Disease Study, depression prevalence in the United States ranges from 11 to 21% and becomes the leading cause of disability-adjusted life-years (DALYs) worldwide [6, 7]. Due to the high burden of the two diseases, more attention has been paid to explore the potential association between periodontitis and depression and further prevent their comorbids in primary care.

At present, there were a large number of studies, including epidemiological evidence and the potential biological mechanisms, demonstrating the linkage between periodontitis and depression [4, 5, 8]. In addition, there was an overlap between risk factors for periodontitis and depression, including smoking, drinking, physical activity, and poor oral hygiene practices [4]. Further evidence indicated that cigarette smoking was responsible for periodontitis [9], and it also can predict the severity and progression of depression [8, 10]. Similarly, drinking also has a minor but significant association with periodontal health, and heavy drinking frequently co-occurs with depression [11, 12]. Considering the fact that periodontitis and depression are both multifactorial and co-occurring disorders [13], further investigation into the relationship of smoking, drinking and depression with periodontitis risk, may help understand periodontitis and its comorbids conditions and further contribute to the prevention of periodontitis and depression.

Interaction analysis is a useful approach for examining multiple modifiable exposure factors that play a role simultaneously or interact with one another on disease risk [14]. It has been widely used in gene–gene and gene-environment studies but less frequently used in periodontitis. According to recent literature, smokers with stage III periodontitis had increased stress than non-smokers [15]. Similarly, patients with chronic periodontitis, smoking habits and depression had the highest levels of salivary cortisol levels [16]. It has also been reported that populations with both drinking and smoking had higher risk for developing periodontitis than those with either factor alone, indicating that they have interactive effects on periodontitis risk [17]. Despite the importance of understanding how these factors can interact on periodontitis risk, most previous epidemiological studies have focused only on single health behavior in isolation. There is no comprehensive interaction analysis on multiple lifestyle factors. Therefore, it is of clinical significance to investigate the interactive association between these reversible health risk factors and periodontitis.

This study aimed to investigate the independent and interactive effects of smoking, drinking and depression on the prevalence of periodontitis based on the National Health and Nutrition Examination Survey (NHANES, 2009–2014).

Null Hypothesis was: There is no significant influence, either individually or collectively, of smoking, drinking, and depression on the risk of periodontitis prevalence.

## Methods

### Study design and populations

**Fig. 1:**
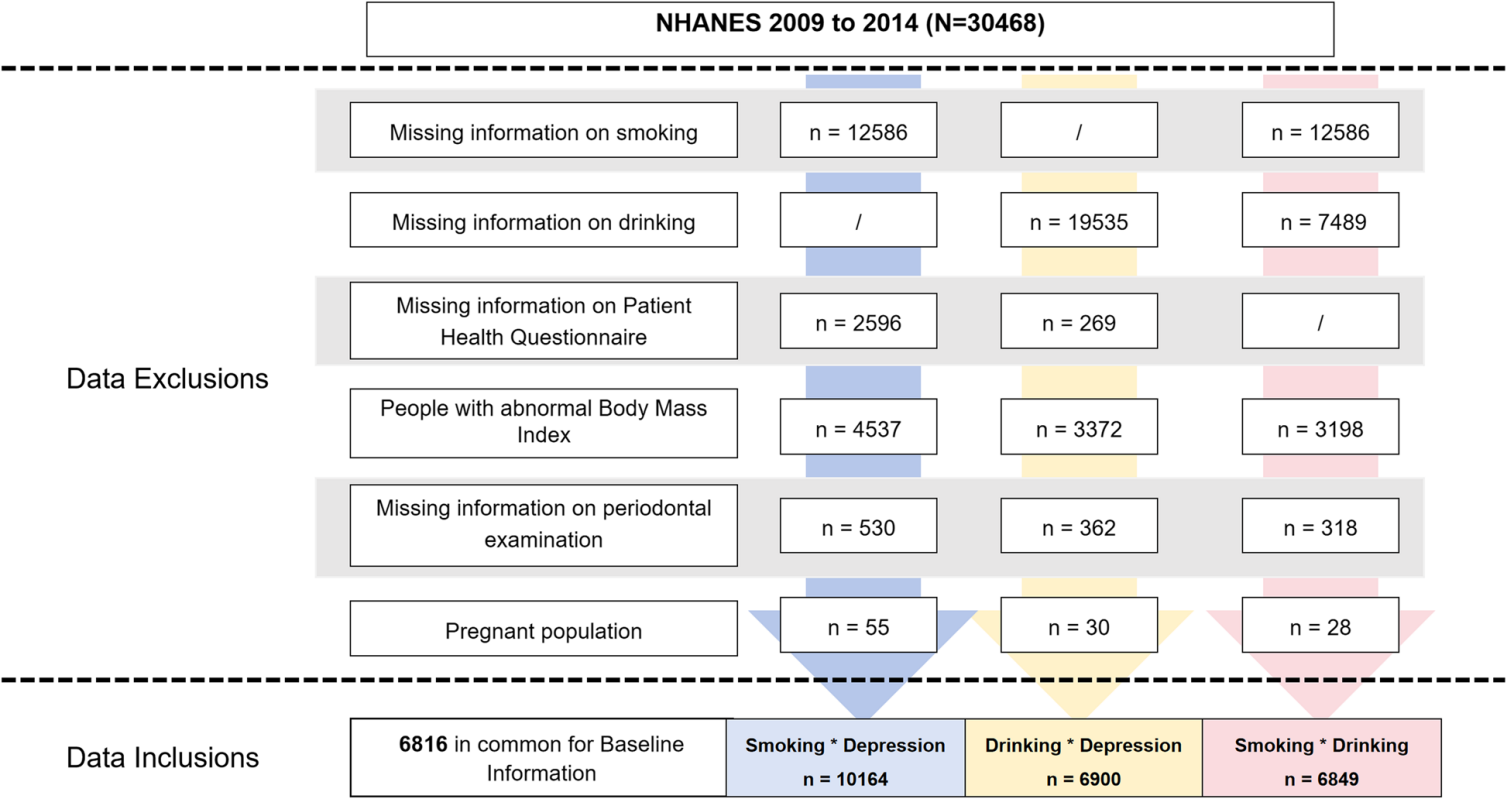
Flowchart of the sample selection from NHANES 2009–2014

NHANES is a comprehensive continuous cross-sectional study conducted in the United States designed to evaluate the nutritional status and overall well-being of the U.S. population. To achieve this, a sample of approximately 5,000 non-institutionalized civilians, selected through a stratified multistage probability sampling method, is recruited on an annual basis to ensure national representation. Data from the NHANES 2009–2014 were included in this study. Participants were invited to complete a detailed home interview and a health examination in a mobile examination center (MEC), including a periodontal examination (age ≥ 30 years) and a depression screening (age ≥ 18 years) [18]. All NHANES protocols that generated the data were approved by the National Center for Health Statistics (NCHS) at the Centers for Disease Control and Prevention (CDC). A total of 30,468 participants from the selected NHANES cohorts were divided into three groups in our analysis. Data inclusion process is shown in Fig. 1. The study excluded participants who had incomplete data on exposure variables including smoking, alcohol consumption, or depression, as well as those who did not undergo periodontal examinations, had abnormal BMI (including absent BMI and extreme BMI values), or were pregnant. Finally, we screened the following three groups as the smoking-depression group (10,164 individuals), drinking-depression group (6,900 individuals, alcohol consumption), and drinking-smoking group (6,849 individuals, the frequency of drinking).

### Periodontitis assessment (outcomes)

All oral health assessments were conducted within a specifically designated room at the mobile examination center (MEC), equipped with a portable dental chair, light, and compressed air. Trained dentists performed the periodontal examination, measuring clinical attachment loss (CAL) and probing depth at six interproximal sites per tooth (distal-facial, mesial, proximal, distal-lingual, mesial-lingual, and proximal-lingual), excluding third molars, and then a health technician entered the collected data. Only individuals aged 30 years and above were considered eligible for participation in these periodontal examinations. The proportion of sites with a CAL ≥ 3 mm is commonly used as an indicator of the severity of periodontitis. In our study, we identified periodontitis in each subject with at least one site exhibiting a CAL ≥ 3 mm [19, 20].

### Depression, drinking, smoking (exposures)

Depression was assessed with the Patient Health Questionnaire-9 (PHQ-9), including nine items about the frequency of depressive symptoms over the previous two weeks. PHQ-9 total scores ranged from 0 to 27 and the cutoff score for depression identification was 10 or more [21]. The drinking and smoking data were collected by computer-assisted personal interview (CAPI) technology. Daily alcohol consumption in the past 12 months was categorized into three levels: low-intensity (1 drink or less), moderate (between 1 and 3 drinks), heavy drinking (4 drinks or more) [22]. A ‘drink’ was defined as containing 14g alcohol. The frequency of weekly alcohol consumption was categorized as ‘5 days or more per week’ (regular drinkers), ‘between 2 and 4 days per week’ (occasional drinkers), and “1 day or less per week’ (non-drinkers). Smokers were defined as those who reported smoking at least 100 cigarettes during their lifetime, either currently (current smoker) or not currently (former smoker) [18].

### Covariates assessment

The sociodemographic variables included in this study were age (in years), gender (male, female), race/ethnicity (non-Hispanic white, non-Hispanic black, non-Hispanic Asian, Mexican Americans, and other race), education level (less than 9th Grade, 9-11th Grade, high school grade/ General Education Development or Diploma or equivalent, some college or Associate of Arts Degree, college graduate or above, missing), marital status (married/living with partner, widowed/divorced/separated, missing), history of diabetes (no, yes, missing), and family monthly poverty level (≤ 1.30, 1.30–1.85, 1.85–5, > 5, missing) [23, 24], as per the poverty guidelines outlined by the U.S. Department of Health and Human Services (HHS) and the recommendations provided by the National Health and Nutrition Examination Survey (NHANES) [25]. Body mass index (BMI) was classified as low weight (< 18.5 kg/m^2^), normal weight (18.5 to 25 kg/m^2^), overweight (25 to 30 kg/m^2^) and obesity (≥ 30 kg/m^2^) [26]. Diagnosed diabetes mellitus, damaging the healing process of periodontitis, was identified by self-report questionnaires [21].

### Statistical analysis

Considering the complicated sample design, all analyses incorporated the NHANES sampling weights to ensure the representativeness of noninstitutionalized civilian resident population of the United States. Descriptive data were presented as mean (standard deviation) for continuous variables and number (percentage) for categorical variables in terms of sociodemographic and periodontal characteristics. The multivariate logistic regression model was used to analyze the independent effects and multiplicative interaction between depression, smoking, and drinking based on initial model and adjusted model (adjusted for age, gender, ethnicity, education, household income and history of diabetes). We analyzed the interaction effect across different gender (male and female) and age groups (≤ 45 and > 45) [27]. Odds ratio (OR) and 95% confidence interval (CI) were calculated. All statistical analyses were performed using the SPSS 24.0 statistical software package (IBM SPSS Statistics for Windows, Version 24.0) and R (versionversion 4.3.1). The statistical significance was set at *P* < 0.05 using the two-sided test.

## Results

### Demographic and periodontal characteristics

Baseline information, including demographic characteristics, physiological status, periodontal status, smoking and drinking levels has been presented in Table 1. Among the 6816 participants included in this study, 55.8% were male and 44.3% were female; 49.4% were non-smokers and 50.6% were smokers (including former and current smokers); 14.7% were low drinkers, 69.9% were moderate drinkers, and 15.4% were heavy drinkers. In the total population, the prevalence of periodontitis and depression was 38.8% and 7.9%, respectively.

**Table 1:** Demographic characteristics, physiological status, smoking and drinking levels: NHANES (2009 to 2014)^a^

Characteristics	Total population	No periodontitis	Periodontitis
	**(*N*** = 6,816)	**(*N*** = 4,171)	**(*N*** = 2,645)
**Age (years), Mean ± SD**	51.52 (14.10)	53.30 (13.78)	48.72 (14.15)
**Depression status, n (%)**			
No depression	6280(92.1)	3848(92.*3*)	2432(9*1.9***)**
Depression	536(7.*9***)**	323(7.7)	213(8.*1*)
**Gender, n(%)**			
Male	3800 (55.*8***)**	2597 (62.*3***)**	1203 (45.*5***)**
Female	3016 (*44.2***)**	1574 (37.7)	1442 (54.5)
**Ethnicity, n (%)**			
Mexican American	885 (*13.0***)**	654 (15.*7***)**	231 (8.7)
Other Hispanic	659 (9.*7***)**	383 (9.*2***)**	276 (10.4)
Non-Hispanic White	3304 (48.*5***)**	1825 (43.*8***)**	1479 (55.9)
Non-Hispanic Black	1292 (1*9.0***)**	901 (21.6)	391 (14.*8***)**
Other Race-Including Multi-Racial	676 (9.9)	408 (9.*8***)**	268 (10.1)
**Marital status, n (%)**			
Married/Living with Partner	4463 (65.*5***)**	2684 (64.*3***)**	1779 (67.*3***)**
Widowed/Divorced/Separated	2350 (34.*5***)**	1484 (35.*6***)**	866 (32.*7***)**
Refused or unknown	3 (0.0)	3 (0.*1***)**	0
**BMI** **status, n (%)**			
Underweight (18.5 kg/m2**)**	73 (1.*1***)**	44 (1.*1***)**	29 (1.*1***)**
Normal (18.5–25 kg/m2**)**	1840 (27.*0***)**	1047 (25.*1***)**	793 (*30.0***)**
Overweight (25–30 kg/m2**)**	2544 (37.3)	1559 (37.*4***)**	985 (37.2)
Obese (> 30 kg/m2**)**	2359 (34.6)	1521 (36.*5***)**	838 (31.*7***)**
**Education, n (%)**			
Less than 9th Grade	497 (7.*3***)**	375 (*9.0***)**	122 (4.6)
9-11th Grade	871 (12.*8***)**	604 (14.*5***)**	267 (10.*1***)**
High School grade/ GED or Equivalent	1453 (21.3)	974 (23.*4***)**	479 (18.1)
Some College or AA Degree	1959 (28.7)	1190 (28.5)	769 (29.*1***)**
College Graduate or above	2030(29.*8***)**	1024 (24.*6***)**	1006 (38.0)
Refused or unknown	6 (0.*1***)**	4 (0.*1***)**	2 (0.*1***)**
**Family monthly poverty level index, n (%)**			
≤ 1.30	1642 (24.*1***)**	1138 (27.*3***)**	504 (19.*1***)**
1.30–1.85	695 (10.*2***)**	482 (11.*6***)**	213 (8.*1***)**
1.85–5	2450 (*36.0***)**	1463 (35.*1***)**	987 (37.*3***)**
> 5	1516 (22.*2***)**	773 (18.*5***)**	743 (28.*1***)**
Refused or unknown	513 (7.*5***)**	315 (7.*6***)**	198 (7.*5***)**
**Smoking status, n (%)**			
Never smoked	3369 (49.4)	1890 (45.3)	1479 (55.9)
Form smoker	1890 (27.7)	1200 (28.8)	690 (26.*1***)**
Current smoker	1557 (22.8)	1081 (25.9)	476 (18.0)
**Drinking status, n (%)**			
**Amount of drinking**			
0–14 g/d	1004 (14.7)	597 (14.3)	407 (15.*4***)**
14–56 g/d	4766 (69.9)	2828 (67.8)	1937 (73.*2***)**
> 56 g/d	1047 (15.*4***)**	746 (17.*9***)**	301 (11.*4***)**
** Frequency of drinking, n(%)**			
Once or less/week	4391 (64.4)	2637 (63.2)	1754 (66.3)
2–4 times/week	1803 (26.*5***)**	1100 (26.*4***)**	703 **(26.***6***)**
> 5 times/week	622 (9.1)	434 (10.4)	188 **(7.1)**
**History of diabetes, n (%)**			
No	5948 (87.*3***)**	3586 (*86.0***)**	2362 (89.3)
Yes	721 (10.*6***)**	495 (11.*9***)**	226 (8.5)
Refused or unknown	147(2.*2***)**	90 (2.*2***)**	57 (2.*2***)**

^a^Descriptive data were shown as mean (standard deviation) for continuous variables and number (percentage) for categorical variables

*N* number, *SD* standard deviation, *BMI* Body Mass Index, *GED* General Education Development or Diploma, *AA* Associate of Arts

### Independent effect of smoking, drinking and depression on periodontitis

As shown in Supplemental Fig. 1, the ORs for developing periodontitis for former and current smokers were 1.23 (95% CI: 1.05 to 1.43) and 1.66 (95% CI: 1.34 to 2.05) when compared with non-smokers in the multivariable-adjusted regression model with all variables. For regular and occasional drinkers, the ORs were 1.14 (95% CI: 0.94 to 1.37) and 1.38 (95% CI: 1.04 to 1.83), respectively. The restricted cubic spline further revealed that drinking had decreased the risk of periodontitis under a low-dose condition but increased the risk under a high-dose condition (*P* < 0.05). The logistic regression model observed no statistically significant association between depression and periodontitis (*P* > 0.05).

Interactive effects of smoking and depression on periodontitis.

**Fig. 2:**
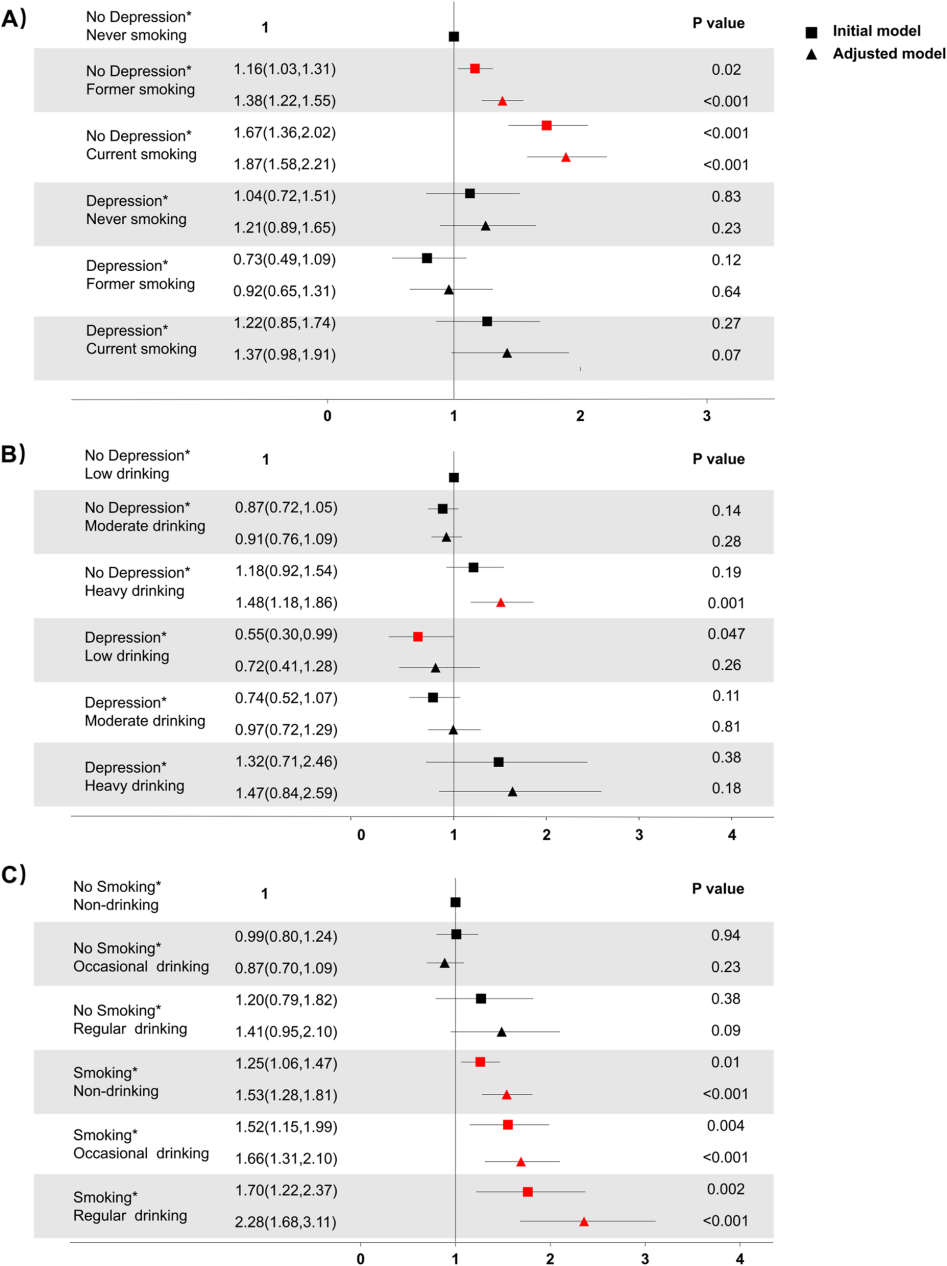
Association between smoking-drinking-depression and the risk of periodontitis. **A** OR of periodontitis in the smoking-depression group; (**B**) OR of periodontitis in the drinking-depression group; (**C**) OR in of periodontitis in the drinking-smoking group. *P*-values less than 0.05 (*P* < 0.05) were considered significant. *OR* odds ratio, *CI* confidence interval, *N* number

There was a significant multiplicative interaction between smoking and depression in the total sample (*P* < 0.05; Table 2). And this interaction was also significant in the male (*P* < 0.05). The subgroup analyses revealed that former smokers (OR: 1.16, 95% CI: 1.02 to 1.31) and current smokers (OR: 1.67, 95% CI: 1.37 to 2.02) had higher risk for developing periodontitis than non-smokers in the non-depressed population (PHQ-9 score < 10, Table S1). Stratified analyses further showed that participants with both smoking and depression had 37% higher prevalence of periodontitis than those without smoking or depression, but this was not statistically significant (OR: 1.37, 95% CI: 0.98 to 1.91; Fig. 2A).

**Table 2:** Interaction of smoking, drinking and depression on periodontitis stratified by age and gender

*P* interaction	**N**	**Total Population**	**Male**	**Female**	**Age ≤ 45**	**Age > 45**
Depression*Smoking	10,164	0.03^*^	0.03^*^	0.35	0.65	0.11
Depression*Drinking	6900	0.16	0.74	0.08	0.78	0.07
Smoking*Drinking	6849	0.39	0.95	0.05^*^	0.60	0.25

^*^indicates *P* < 0.05. The adjusted model was adjusted for age, gender, ethnicity, family income-to-poverty ratio, educational level, and history of diabetes.* P*-values less than 0.05 (*P* < 0.05) were considered significant

*OR* odds ratio, *CI* confidence interval, *N* number

### Interactive effects of drinking and depression on periodontitis

In the total population and subgroups, no significant multiplicative interaction (*P* > 0.05) was identified between alcohol consumption and depression on periodontitis risk (Table 2). Stratified analysis further demonstrated that when combined with depression, heavy drinkers increased prevalence of periodontitis by 47%, but this was not statistically significant (OR: 1.47,95% CI: 0.84 to 2.59; Fig. 2B), compared to the general population without drinking or depression.

### Interactive effects of smoking and frequency of drinking on periodontitis

There was a significant multiplicative interaction between smoking and drinking in the female population (*P* < 0.05; Table 2). When combined with smoking status, the risk for developing periodontitis increased with an increased frequency of drinking (for non-drinkers, OR: 1.53, 95% CI:1.28 to 1.81; for occasional drinkers, OR:1.66, 95% CI:1.31 to 2.10; for regular drinkers, OR: 2.28, 95% CI:1.68 to 3.11; Fig. 2C).

## Discussion

This is the first systematic examination of the interaction between depression, smoking, and alcohol consumption on the prevalence of periodontitis using a large US population. In the overall population and within gender subgroups, we observed significant multiplicative interactions between smoking-depression and smoking-drinking. In the depressed population, smoking significantly increased the prevalence of periodontitis by 1.29-fold in the youth group compared with non-smoking. Moreover, the co-occurrence of smoking and heavy alcohol consumption exhibited a significant elevation in the prevalence of periodontitis. Based on the findings of this study, our null hypothesis was *rejected*.

Our study found that cigarette smoking served as a risk factor for periodontal diseases, as previously reported [9]. Results showed that both former and current smokers were more likely to develop periodontitis than non-smokers in the general population (PHQ-9 score < 10). The proposed explanation was that smoking effects on inducing systemic inflammation can continue for months or years despite the fact that cigarette smoke extracts have been removed from the body after smoking cessation [9]. This result was inconsistent with previous studies, which indicated no significant difference between never-smokers and former smokers regarding periodontitis risk [28, 29]. The heterogeneity of research can be attributed to different time frames for evaluating smoking status. It has been further reported that until approximately 6 years after quitting smoking, the smoking-related risk for periodontitis will diminish and approach to that of never-smokers [30].

To our knowledge, this is the first time that the interaction between smoking and depression has been indicated in periodontitis. Previous evidence have demonstrated that psychological stress and smoking may serve as mediators for the prevalence of periodontal diseases [31]. Both biological mechanisms, such as inflammatory responses [9] and behavioral theories have been proposed to explain how smoking and depression independently influenced periodontitis. The mechanisms underlying the correlations between smoking and periodontitis shared similarities with those linking depression to periodontitis [9]. One proposed explanation for this synergistic effect is that smokers with a history of depression can have a prolonged and/or augmented inflammatory response since the anti-inflammatory response capability may be downregulated by depression condition. Then, a hyper-inflammatory response can be reinforced by smoking status, thereby accelerating the progression of periodontitis [9]. Considering our findings, more attention should be paid to the screening and prevention of periodontitis in depression subjects and depression in periodontitis patients by collecting comprehensive information regarding the smoking history and depressive symptoms.

Further results showed that the smoking-depression interaction exhibited consistency across various age and gender cohorts. This interaction was only significant in males not females due to the exceptionally low smoking rates among women in this study. Additionally, depressed men frequently failed to maintain healthy behaviors, such as smoking cessation [32]. On the other hand, this association was consistent in both young and aged populations. Therefore, regardless of an individual's age, smoking consistently has adverse impacts on periodontitis, regardless of the duration of smoking, whether it is of a short-term or long-term nature.

A dose-dependent relationship was identified between drinking and periodontitis, as well as between smoking-drinking interaction and periodontitis. This finding aligns with previous research, indicating the association between cigarette smoking and increased periodontitis risk with alcohol assumption increment [17]. The potential hypothesis is that the dysfunction of immune cells, specifically T-cells and neutrophils, may be responsible for the cumulative effects observed with excessive alcohol intake surpassing a specific threshold [33]. Subsequently, the risk for infectious complications including periodontitis can be elevated due to the excessive alcohol intake [11]. Overall, these results underscore the adverse effects of alcohol consumption itself, as well as its confounding adverse effects when combined with other factors such as smoking on periodontitis progression. This highlights the significance of limiting cumulative alcohol intake from both the individual and combined effects.

Our study has some strengths. First, we analyzed a large-scale population and examined the complicated interactive relationship between different influencing factors rather than focus on only one single factor. Second, data from the NHANES were selected using a complex, multi-stage probability sampling design fully representative of the general population across the United States. Third, multiple confounding factors were considered in this study, including sociodemographic characteristics and history of diabetes. There are some limitations to this study. First, no causality can be inferred from the data due to the cross-sectional nature of the study. As with other cross-sectional studies, the temporal relationship cannot be established considering the long-term variables (e.g., periodontitis, smoking) and the short-term variables (e.g., drinking and depression). Therefore, further study is needed to solve geographic restrictions from a large multinational population. Second, our study utilized CAL as the sole criterion for grading the severity of periodontitis, without considering other factors like aggressive tooth brushing and periodontal trauma. This may lead to some bias in the patients inclusion, which could be improved by designing comprehensive and detailed epidemiological surveys. However, it is important to consider that such improvements often come with higher survey costs and longer research cycles, therefore a balance needs to be achieved between the accuracy, cost, and time in the future survey designs.

## Conclusion

In summary, there was a significant multiplicative interaction between smoking-depression and smoking-drinking, and promoting healthy behaviors and mental well-being may play a crucial role in the prevention of periodontitis.

### Supplementary Information

**Additional file 1:** **Figure S1.** Association between individual lifestyle factor and the risk of periodontitis. A) Association between smoking and periodontitis; B) Association between drinking frequency and periodontitis risk; C) Association between drinking amount and periodontitis risk. Model was adjusted for age, gender, ethnicity, family income-to-poverty ratio, educational level, and history of diabetes. *P*-values less than 0.05 (*P *< 0.05) were considered significant. OR: odds ratio, CI: confidence interval.

**Additional file 2:** **Table S1. **The interaction of smoking, drinking and depression on periodontitis stratified by age and gender.

## Data Availability

Publicly available datasets were analyzed in this study. Data for this study are available at https://www.cdc.gov/nchs/nhanes/index.htm.

## Abbreviations

NHANES: National Health and Nutrition Examination Survey
OR: Odd ratio
CI: Confidence interval
NCHS: National Center for Health Statistics
CDC: Centers for Disease Control and Prevention
DALYs: Disability-adjusted life-years
PD: Periodontal probing depth
CAL: Clinical attachment loss
MEC: Mobile examination center
PHQ-9: Patient Health Questionnaire-9
CAPI: Computer-assisted personal interview
BMI: Body mass index
RCS: Restricted cubic spline
