# Supplementary figures and images for: Independent and combined effects of smoking, drinking and depression on periodontal disease
Source: BMC Oral Health. 2024 May 6;24:535. doi: 10.1186/s12903-024-04287-6 (PMC11075253; doi:10.1186/s12903-024-04287-6)

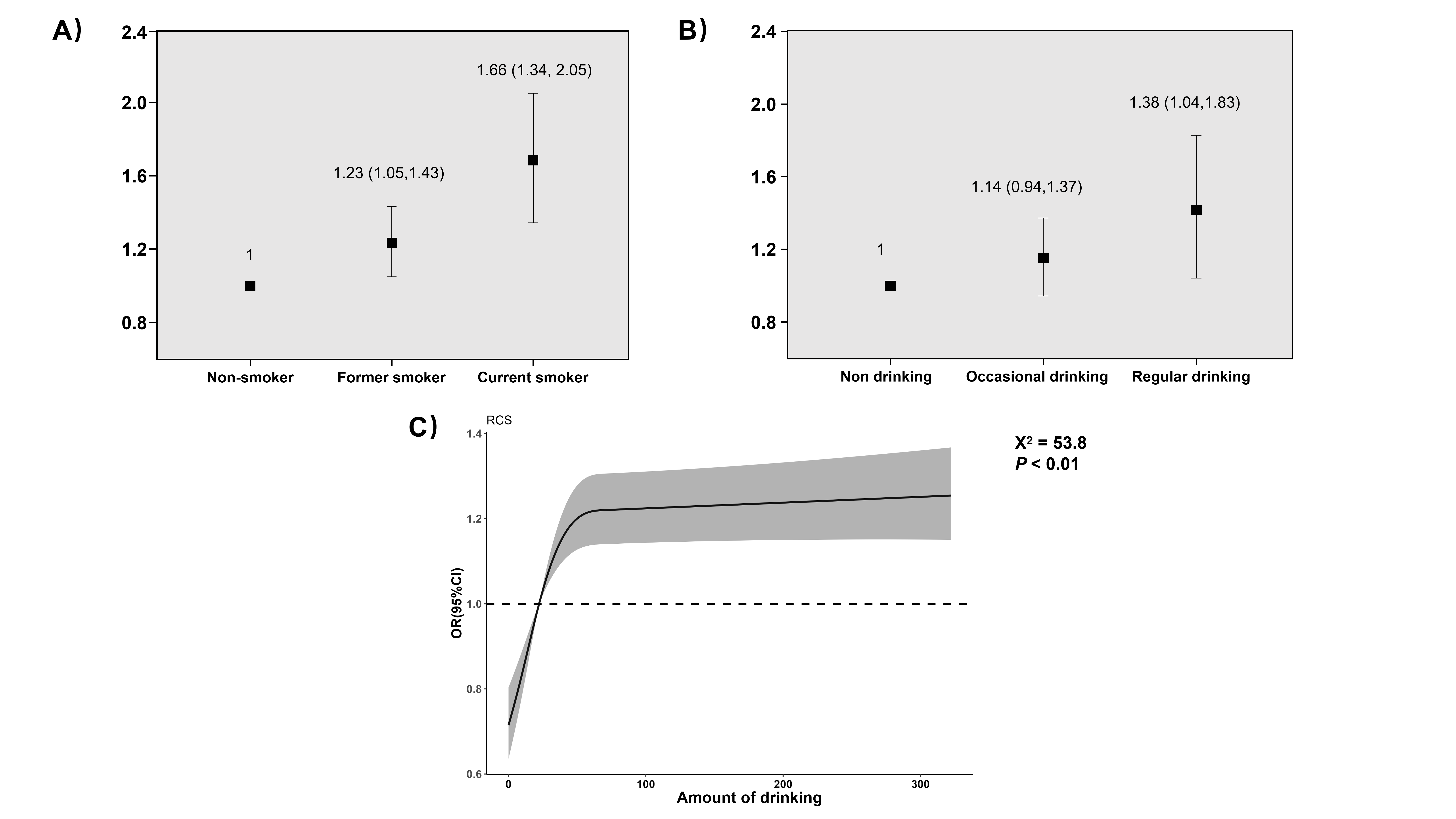

Supplement: Supplementary file 1 — Additional file 1: Figure S1. Association between individual lifestyle factor and the risk of periodontitis. A) Association between smoking and periodontitis; B) Association between drinking frequency and periodontitis risk; C) Association between drinking amount and periodontitis risk. Model was adjusted for age, gender, ethnicity, family income-to-poverty ratio, educational level, and history of diabetes. P-values less than 0.05 (P < 0.05) were considered significant. OR: odds ratio, CI: confidence interval. [file 12903_2024_4287_MOESM1_ESM.tif]
