# Supplementary material for: Independent and combined effects of smoking, drinking and depression on periodontal disease
Source: BMC Oral Health. 2024 May 6;24:535. doi: 10.1186/s12903-024-04287-6 (PMC11075253; doi:10.1186/s12903-024-04287-6)
Supplement: Supplementary file 2 — Additional file 2: Table S1. The interaction of smoking, drinking and depression on periodontitis stratified by age and gender. [file 12903_2024_4287_MOESM2_ESM.docx]

**Table S1** The interaction of smoking, drinking and depression on periodontitis stratified by age and gender.

| **Subgroups** |  |  | Total Population | Male | Female | Age ≤ 45 | Age > 45 |
| --- | --- | --- | --- | --- | --- | --- | --- |
|  |  |  | **OR (95%CI)** | **OR (95%CI)** | **OR (95%CI)** | **OR (95%CI)** | **OR (95%CI)** |
| **Depression*Smoking** |  | **N** | **P interaction =** 0.03* | **P interaction =** 0.03* | **P interaction =** 0.35 | **P interaction =** 0.65 | **P interaction =** 0.11 |
| No depression | Never smoking | 5045 | Ref | Ref | Ref | Ref | Ref |
|  | Former smoking | 2507 | 1.16 (1.02-1.32) * | 1.13 (0.94-1.36) | 1.20 (0.98-1.46) | 1.21(0.93-1.56) | 1.17 (0.99-1.39) |
|  | Current smoking | 1728 | 1.67 (1.37-2.03) *** | 1.64 (1.24-2.16) *** | 1.72 (1.26-2.34) *** | 1.81 (1.34-2.43) *** | 1.50 (1.15-1.95) ** |
| Depression | Never smoking | 349 | Ref | Ref | Ref | Ref | Ref |
|  | Former smoking | 218 | 0.72 (0.49-1.06) | 0.44 (0.19-1.00) | 1.03 (0.59-1.82) | 0.70 (0.27-1.84) | 0.63 (0.37-1.08) |
|  | Current smoking | 317 | 1.17 (0.65-2.09) | 1.19(0.46-3.08) | 1.13 (0.58-2.22) | 2.29 (1.10- 4.76) * | 0.69 (0.32-1.49) |
| **Depression*Drinking** |  | **N** | **P interaction =** 0.16 | **P interaction =** 0.74 | **P interaction =** 0.08 | **P interaction =** 0.78 | **P interaction =** 0.07 |
| No depression | Low drinking | 1004 | Ref | Ref | Ref | Ref | Ref |
|  | Moderate drinking | 4428 | 0.86 (0.71-1.05) | 0.86 (0.67-1.11) | 0.87 (0.67-1.14) | 1.11 (0.81-1.53) | 0.80 (0.62-1.03) |
|  | Heavy drinking | 925 | 1.11 (0.84-1.47) | 1.27 (0.90-1.77) | 0.80 (0.53-1.22) | 1.60 (1.04- 2.48) * | 0.93 (0.64-1.35) |
| Depression | Low drinking | 68 | Ref | Ref | Ref | Ref | Ref |
|  | Moderate drinking | 352 | 1.43 (0.80-2.58) | 1.12 (0.37-3.43) | 1.86 (0.82-4.22) | 2.04 (0.77-5.43) | 1.47 (0.68-3.17) |
|  | Heavy drinking | 123 | 1.99 (0.81-4.91) | 1.88 (0.42-8.46) | 2.07 (0.59-7.30) | 2.48 (0.97- 6.29) | 1.84 (0.43-7.84) |

* indicates *P* <0.05, ** indicates *P* <0.01, *** indicates *P* <0.001. The adjusted model was adjusted for age, gender, ethnicity, family income-to-poverty ratio, educational level, and history of diabetes. *P*-values less than 0.05 (*p* < 0.05) were considered significant. OR: odds ratio; CI: confidence interval. N, number.
